# The NF-κB RelB Protein Is an Oncogenic Driver of Mesenchymal Glioma

**DOI:** 10.1371/journal.pone.0057489

**Published:** 2013-02-25

**Authors:** Dong Whan Lee, Dhivya Ramakrishnan, John Valenta, Ian F. Parney, Kayla J. Bayless, Raquel Sitcheran

**Affiliations:** 1 Department of Molecular & Cellular Medicine, Texas A&M University Health Science Center, College Station, Texas, United States of America; 2 Department of Neurologic Surgery, Mayo Clinic Cancer Center, Rochester, Minnesota, United States of America; Duke University, United States of America

## Abstract

High-grade gliomas, such as glioblastomas (GBMs), are very aggressive, invasive brain tumors with low patient survival rates. The recent identification of distinct glioma tumor subtypes offers the potential for understanding disease pathogenesis, responses to treatment and identification of molecular targets for personalized cancer therapies. However, the key alterations that drive tumorigenesis within each subtype are still poorly understood. Although aberrant NF-κB activity has been implicated in glioma, the roles of specific members of this protein family in tumorigenesis and pathogenesis have not been elucidated. In this study, we show that the NF-κB protein RelB is expressed in a particularly aggressive mesenchymal subtype of glioma, and loss of RelB significantly attenuated glioma cell survival, motility and invasion. We find that RelB promotes the expression of mesenchymal genes including YKL-40, a marker of the MES glioma subtype. Additionally, RelB regulates expression of Olig2, a regulator of cancer stem cell proliferation and a candidate marker for the cell of origin in glioma. Furthermore, loss of RelB in glioma cells significantly diminished tumor growth in orthotopic mouse xenografts. The relevance of our studies for human disease was confirmed by analysis of a human GBM genome database, which revealed that high RelB expression strongly correlates with rapid tumor progression and poor patient survival rates. Thus, our findings demonstrate that RelB is an oncogenic driver of mesenchymal glioma tumor growth and invasion, highlighting the therapeutic potential of inhibiting the noncanonical NF-κB (RelB-mediated) pathway to treat these deadly tumors.

## Introduction

High-grade gliomas (HGGs), the most common primary brain tumors, are resistant to standard treatment strategies, resulting in poor patient prognosis and low survival rates [1]. The extensive cellular and molecular heterogeneity that characterizes gliomas allows for efficient adaptation to different microenvironments and survival of tumor cells that are highly invasive. The identification of distinct glioma subtypes based on gene expression profiling has increased our understanding of the molecular basis of differences in patient survival rate and offers enormous potential for predicting responses to treatment [2]–[4]. For example, it has been observed that tumors with a mesenchymal gene expression signature exhibit more aggressive clinical phenotypes, are highly resistant to therapy, and lead to higher rate of relapse and worse overall outcomes than tumors of the classical, proneural, and neural subtypes [2]. Interestingly, a shift to a mesenchymal phenotype is frequently observed in recurrent tumors from the same patient, regardless of the primary tumor subtype [2]. Thus, the aggressiveness of mesenchymal glioma underscores our need for a better understanding of the molecular pathways that mediate mesenchymal gene expression. However, little is known about the key molecular alterations that are responsible for driving tumor growth and survival in specific subgroups.

Nuclear factor κB (NF-κB) is a family of transcription factors that respond to extracellular signals to regulate a wide range of biological processes, such as cell survival, immune and inflammatory responses [5]. Consequently, aberrant NF-κB activity is frequently observed in many chronic inflammatory diseases, including cancer [6], [7]. NF-κB proteins (RelA/p65, RelB, c-Rel, NFκB1 and NFκB2) share a conserved Rel homology domain (RHD) that mediates dimerization and DNA binding. In the canonical NF-κB signaling pathway, RelA activation occurs through signal-dependent activation of IKKβ, which results in degradation of the inhibitory protein IκBα and nuclear translocation of transcriptionally active RelA/p50 heterodimers. In the noncanonical NF-κB pathway, RelB is activated by IKKα-dependent processing of the inhibitory NFκB2/p100 protein to p52, resulting in transcriptionally active RelB/p52 heterodimers [8]. Although NF-κB signaling has been extensively studied, differential roles and specific functions of individual NF-κB proteins in tumorigenesis are not well characterized. Oncogenic roles for RelB have been described [9], [10], but the functions of RelB in gliomas, or other cancers of the CNS, have not been previously examined. However, the expression of RelB in neural precursor cells during mammalian development [11] is consistent with the possibility that increased RelB activity may be involved in CNS cancers.

In this study, we investigate the role of RelB in glioma tumorigenesis and pathogenesis. We identify a critical role for RelB in controlling mesenchymal gene expression and driving oncogenesis in glioma. Our studies are supported by gene expression profiles of glioma patients, which show that high level of RelB expression is associated with rapid tumor progression and low survival rates. Thus, our findings have important implications for targeting the noncanonical NF-κB pathway as a therapeutic strategy for aggressive mesenchymal glioma.

## Results

### High RelB Expression is Associated with Increased Survival of Glioma Cells

**Figure 1:**
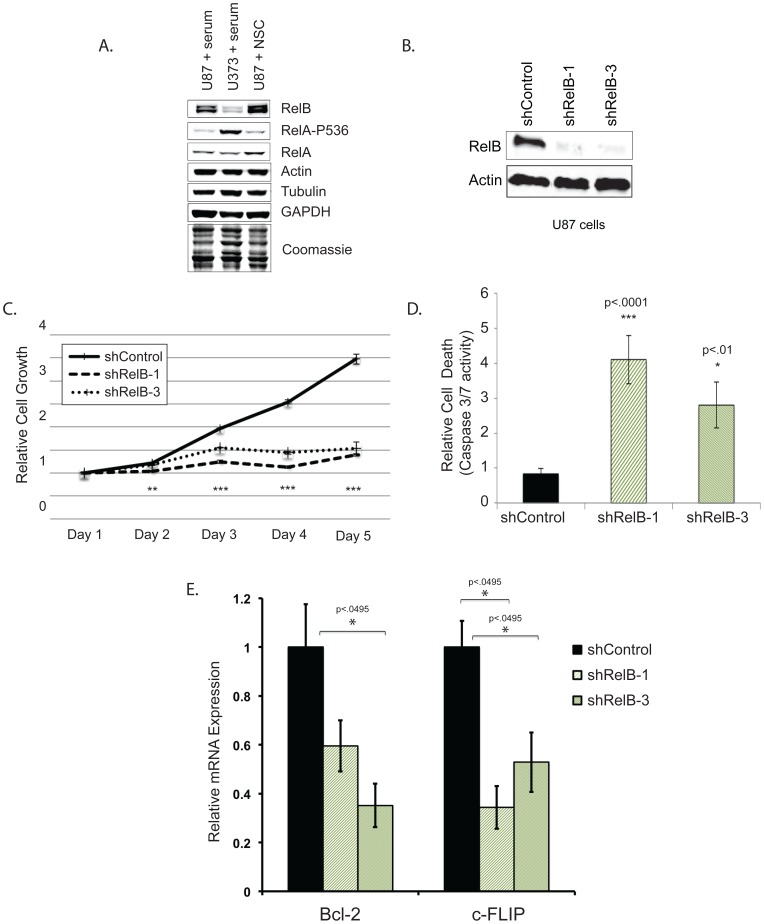
RelB promotes glioma cell proliferation and survival. *(A)* Western blot analysis of glioma cells using indicated antibodies. *(B)* Western blot analysis was used to assess RelB expression in U87 cells stably expressing a scrambled shRNA control or RelB targeting shRNAs. *(C)* MTS assays performed on U87 shRNA control, shRelB-1 and shRelB-3 cell lines. Error bars indicate standard deviation (SD), n = 4. *(D)* A Bioluminescent assay to measure Caspase 3/7 activity was performed on U87 cells expressing the indicated shRNA constructs. Error bars indicate SD. *(E)* Quantitative real-time PCR examining levelsof Bcl-2 and c-FLIP mRNA in RelB knockdown cells. Error bars indicate standard error (n = 3).

It has been reported that the NF-κB transcription factor, RelB, is highly expressed in mesenchymal glioma, compared with the classical, neural and pro-neural glioma subtypes [12]. However, a functional role for RelB in gliomas has not been established. We first evaluated RelB expression levels in two well established glioma cell lines, U87 and U373. When cultured under serum-free conditions in neural stem cell media (NSC), U87 cells grow as non-adherent tumorspheres, contain increased numbers of cancer stem cells (CSCs) [13] and form tumors that more accurately resemble the infiltrative growth of human glioma compared with serum-cultured cells [14]. Using western blot analysis, we found that RelB was expressed at significantly higher levels in U87 compared with U373 cells when grown as adherent cultures in the presence of serum (Figure 1A). In contrast, RelA phosphorylation, a marker of its transcriptional activation, was higher in U373 compared with U87 cells, even though total RelA levels were similar in both cell lines (Figure 1A). U373 cells did not grow proficiently in NSC medium and failed to form tumorspheres (Saltzman and Sitcheran, unpublished).

U87 cells have a mesenchymal gene expression profile similar to that of primary glioblastomas [15] and express high levels of the mesenchymal subtype marker YKL-40 compared with U373 cells [15], [16]. Therefore, we focused our studies on U87 cells and tested whether loss of RelB impacted the growth and survival of glioma tumorspheres. We attenuated RelB expression by transducing U87 cells with two lentiviruses expressing shRNAs targeting different regions of RelB mRNA (shRelB-1 and shRelB-3). Both shRNAs significantly reduced RelB protein levels in U87 cells (Figure 1B), suppressed cell growth and increased caspase-3/7 activity compared with scrambled shRNA controls (Figure 1C, D). Furthermore, expression of the antiapoptotic, RelB-regulated genes Bcl-2 and c-FLIP [10] was significantly reduced in these RelB knockdown cells (Figure 1E). Together, these results suggest that RelB inhibits apoptosis to enhance glioma cell growth and survival. Moreover, these data establish U87 cells as a valid *in vitro* system to manipulate RelB expression and to address its role in mesenchymal gliomagenesis.

### RelB Promotes Glioma Cell Motility and Invasion

**Figure 2:**
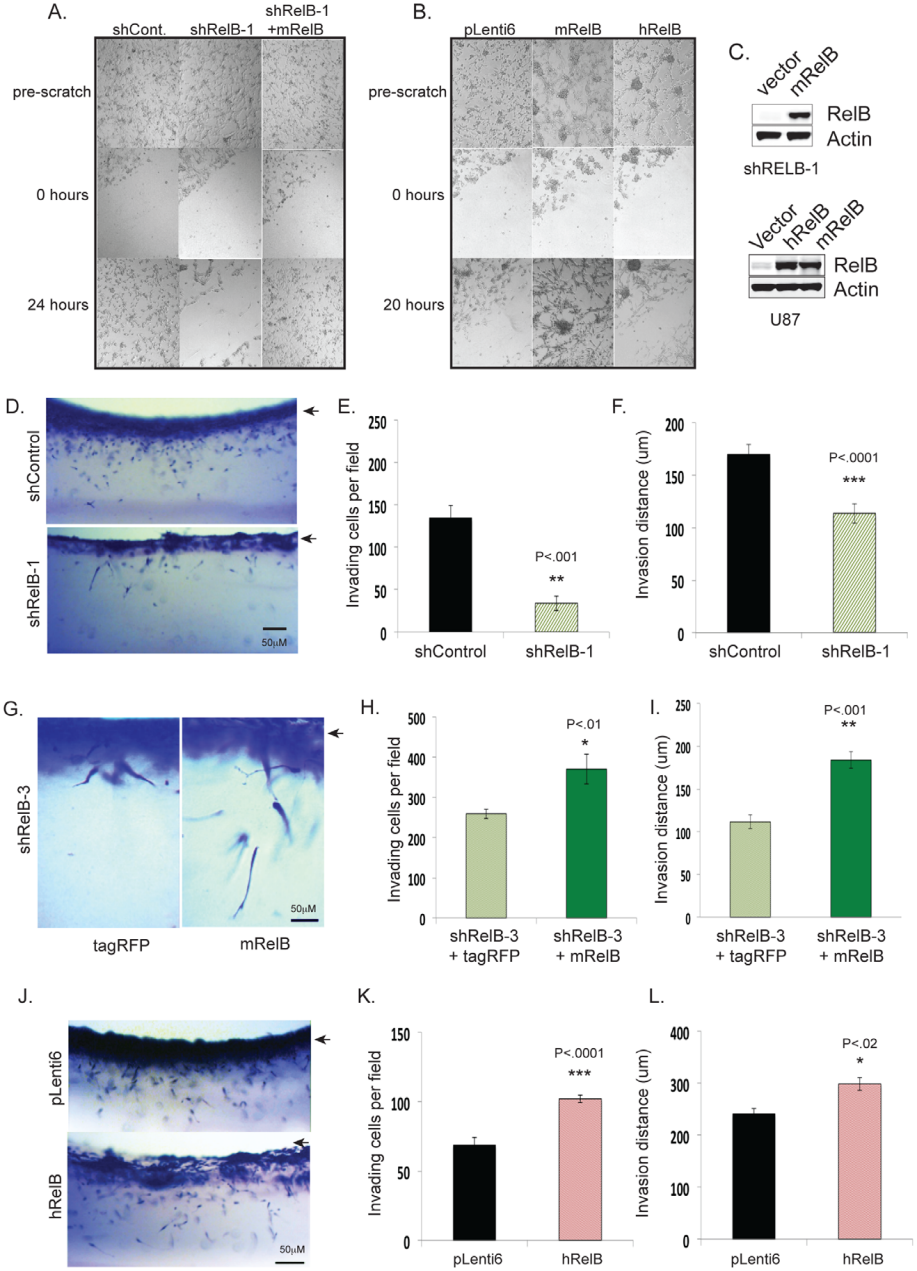
RelB controls glioma cell motility and invasion. *In vitro* scratch assays were performed to compare the motility of *(A)* U87 cells expressing shRNA control, shRelB-1 cells, shRelB-1+vector and shRelB+mRelB; *(B)* U87 cells expressing pLenti6 vector, pLenti6-mRelB, or pLenti6-hRelB. Photographs were taken of cells pre-scratch, 0 hours and 20–24 hours post-scratch. *(C)* Western blot was performed on U87 wild type or shRelB-3 cells using antibodies to RelB and actin. (D*)* Representative photographs of a side view of U87 cells invading three-dimensional collagen matrices. Arrow indicates the surface of the collagen matrix. *(E)* Average numbers of invading cells per field from 3 independent fields (+/− SD). *(F)* Average invasion distances (n = 100 cells) +/− SEM. *(G)* Representative photographs of a side view of U87-shRelB cell invasion +/− rescue with mRelB. *(H)* Quantification of number of invading cells from *G*. *(I)* invasion distance from G. Data shown are average numbers of invading cells per field from 3 independent fields (+/− SD). *(J)* Representative photographs of a side view of U87 cells overexpressing hRelB invading collagen matrices. *(K)* Quantification of invasion from J. Data shown are average numbers of invading cells per field from 3 independent fields (+/− SD). *(L)* Invasion distance from panel J.

To assess a role for RelB in glioma cell motility, we employed an *in vitro* scratch assay [17] using U87 cells grown in serum-containing medium to promote growth as adherent monolayers. Control shRNA cells repopulated a scratched monolayer after 24 hours whereas cells expressing RelB knockdown cells did not efficiently migrate into the wounded area (Figure 2A). However, migration was restored when mouse RelB (mRelB) was re-expressed in RelB knockdown cells (Figure 2A). Wild type U87 cells over-expressing mRelB also repopulated the wounded monolayer more efficiently compared with control cells (Figure 2B, C), demonstrating that RelB enhances cell motility. Cells overexpressing human RelB also migrated faster than control cells, but were less efficient at repopulating the wound than mRelB-overexpressing cells (Figure 2B, C). Interestingly, cells overexpressing either mRelB or hRelB formed more sphere-like clusters compared with pLenti vector control cells (Figure 2B).

**Figure 3:**
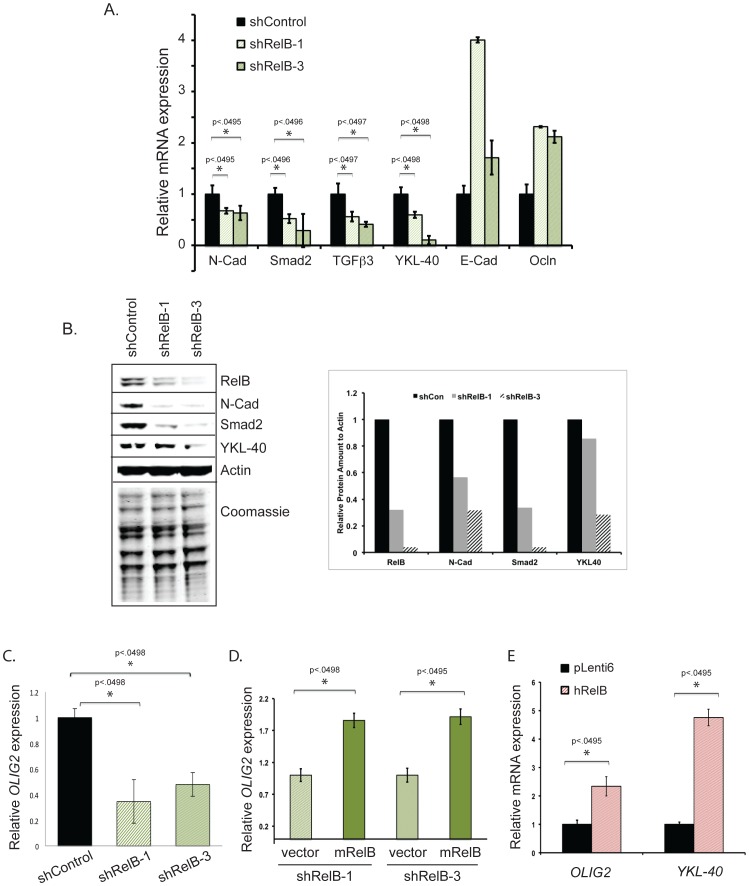
RelB controls the expression of genes associated with EMT. *(A)* Quantitative real-time PCR (qRT-PCR) was performed to analyze expression of the indicated mesenchymal and epithelial genes. *(B)* Western blot analysis with the indicated antibodies was performed on U87 cells expressing control or two independent RelB shRNAs (shRelB-1 and shRelB-3). The accompanying graph shows quantified protein levels in control (shCon) and RelB knockdown cells. *(C)* qRT-PCR analysis of *OLIG2* mRNA levels in shControl and shRelB cells, *(D)* qRT-PCR analysis of *OLIG2* mRNA levels in RelB knockdown cells that contain vector control or rescued mRelB expression. *(E)* qRT-PCR analysis of *OLIG2* and YKL-40 expression in wild type U87 glioma cells that overexpress mRelB.

We next examined glioma cell invasion and migration in three-dimensional (3D) collagen I matrices that maintain cell–cell and cell–extracellular matrix interactions more faithfully than 2D cultures [18], [19]. Significantly fewer RelB knockdown cells invaded 3D collagen-I matrices and migrated a shorter distance compared with control cells (Figure 2D–F). Additionally, overexpression of mRelB in RelB knockdown cells increased both the numbers of invading cells and invasion distance (Figure 3G–I). Overexpression of hRelB in wild type cells also increased invasion density and distance (Figure 3J–L). Together, these results demonstrate that RelB expression strongly correlates with glioma invasion and migration in 3D matrices.

### RelB is a Positive Regulator of *YKL-40* and *OLIG2* Expression

Given the association of high RelB levels with mesenchymal glioma, we next assessed whether RelB controls mesenchymal gene expression. RelB knockdown cells displayed diminished RNA expression of the mesenchymal genes *N-Cadherin/CDH2*, *SMAD2* and *TGFβ3*, as well as lower levels of *YKL-40/CHI3L1* (Figure 3A), a biomarker of aggressive and recurrent gliomas [20], [21] that is very strongly associated with the mesenchymal glioma subtype [2], [3], [15]. Loss of N-cadherin, Smad2 and YKL-40 was also confirmed at the protein level using quantitative western blot analyses (Figure 3B). Conversely, RelB knockdown cells exhibited increased expression of the epithelial cell junction genes *E-Cadherin/CDH1* and *Occludin* (Figure 3A). A main molecular hallmark of epithelial-to-mesenchymal transition (EMT) is a switch in expression from E-cadherin to N-cadherin [22]; thus, these data are consistent with a role for RelB in promoting EMT to control a mesenchymal phenotype in glioma.

In addition to genes regulating EMT, we examined the expression of genes associated with gliomagenesis. *Olig2* is a key regulator of neural progenitor and cancer stem cell proliferation that promotes gliomagenesis [23], [24]. Indeed, U87 cells express *Olig2*, which is significantly downregulated by RelB shRNAs (Figure 3C). Moreover, *Olig2* expression can be rescued in RelB knockdown cells by over-expression of mRelB (Figure 3D), and over-expression of RelB in wildtype U87 cells increased expression of both *Olig2* and *YKL-40* mRNA (Figure 3E).

### RelB Controls Glioma Tumorigenesis *in vivo*

**Figure 4:**
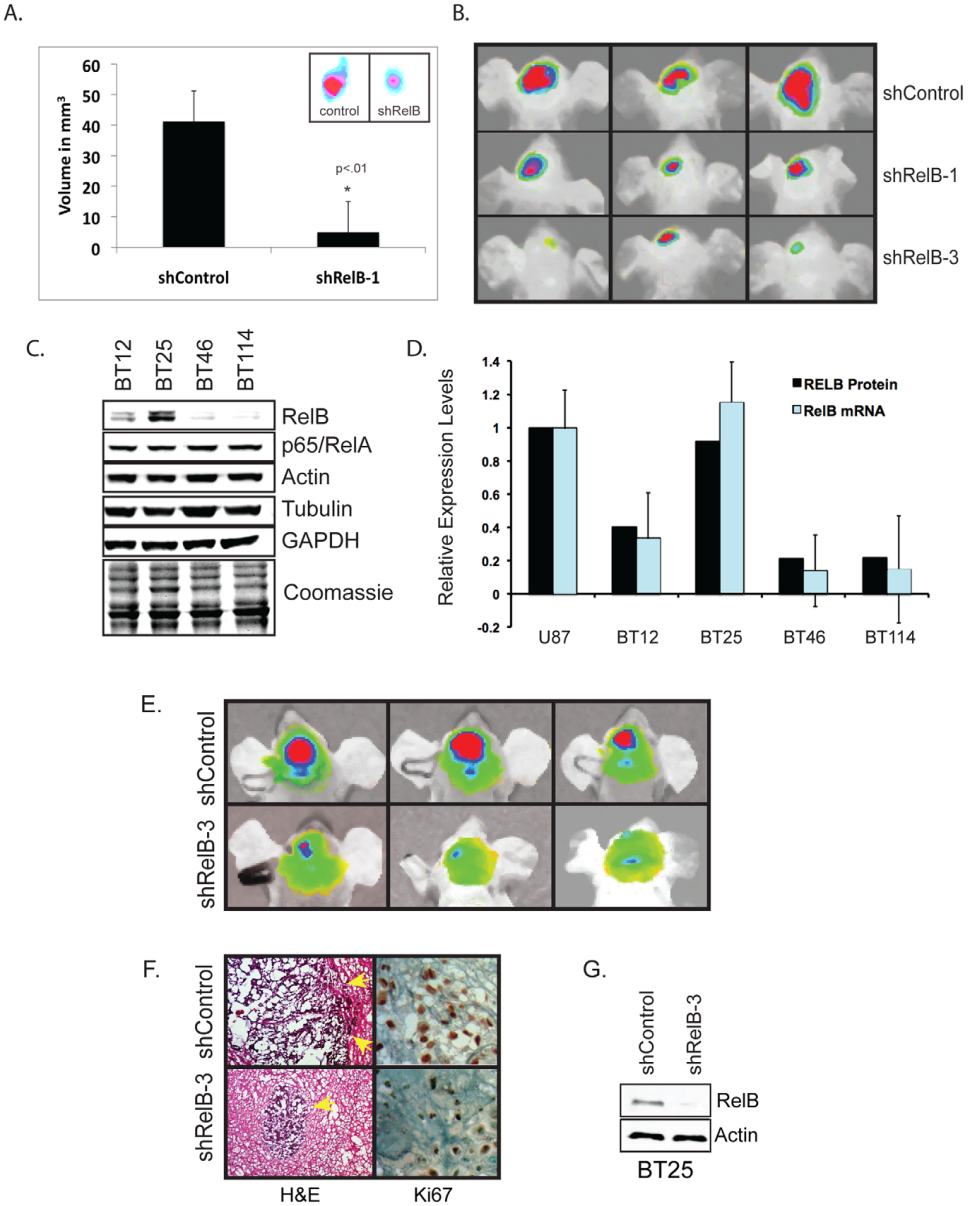
RelB controls tumorigenesis glioma tumorigenesis *in vivo* and is a prognostic indicator of glioma patient survival. *(A)* Subcutaneous xenografts of DiD-labeled U87 shControl and shRelB-1 cells were allowed to grow for 4 weeks (n = 4). Average volume of tumors was determined based on caliper measurement of tumor diameter. Inset shows representative *in vivo* tumor images taken with an *In Vivo* Kodak FX Imager. *(B)* Orthotopic intracranial injection of DiD-labeled U87 shControl, shRelB-1 and shRelB-3 cells were allowed to grow for 4 weeks. Representative *in vivo* tumor images from one experiment are shown (n = 3). *(C)* Western blot analysis was performed on patient-derived glioma cells. *(D)* Comparison of RelB protein and mRNA levels among the indicated cells. To compare RelB protein expression in U87 and BT cells, western blot data from Figs. 1A and 5B were quantified and normalized to Actin. RelB mRNA levels were quantified by real-time PCR. *(E)* Intracranial tumor growth of DiD-labeled BT25 glioma cells expressing shRNA control or shRNA-RelB-3 was evaluated by *in vivo* fluorescence imaging 4 weeks after intracranial innoculation. Representative tumor images from one experiment are shown (n = 3). Similar results were seen with shRelB-1 cells (data not shown). (F) H&E and KI67 staining of frozen brain sections after 4 weeks of tumor growth. Yellow arrows indicate tumor borders. *(G)* Western blot analysis of RelB levels in BT25 shControl and shRelB-3 cells.

To validate the role of RelB in the tumorigenic potential of glioma cells *in vivo,* we assayed tumor growth in subcutaneous and intracranial mouse xenografts using fluorescently labeled tumor cells. We observed that U87 shRelB-1 knockdown cells formed much smaller subcutaneous tumors than U87 shControl cells (Figure 4A). Similarly, orthotopic mouse xenografts with U87 shRelB-1 and shRelB-3 knockdown cells formed much smaller intracranial tumors, compared to U87 shControl cells (Figure 4B).

We next expanded our studies of U87 cells to glioma stem cell explants derived from human patients shown to recapitulate the invasive and diffuse growth in mice [25]. RelB expression varied widely among tumor cells from four different patients, with BT25 cells expressing the highest levels of RelB, comparable to U87 cells (Figure 4C and 4D). Similar to U87 cells, loss of RelB in BT25 cells diminishes cell growth, survival and invasion *in vitro* (Supplemental Figure S3A–D). Additionally, loss of RelB also results in decreased expression of *YKL-40* and *Olig2* (Supplemental Figure S3E, F). Similar to results obtained with U87 cells, BT25 shRelB-3 cells formed significantly smaller intracranial tumors compared to BT25 shControl cells (Figure 4E). Immunohistochemical analysis revealed that BT25 shControl cells formed tumors were very large and highly invasive, with irregular borders, whereas shRelB-3 cells derived tumors were much smaller, with compact, well-defined borders (Figure 4F). Moreover, consistent with in vitro data (Supplemental Figure S3), compared with shControl tumors, BT25 shRelB-3 tumors have diminished expression of the proliferation marker KI67 (Figure 4F). Western blot analysis confirmed efficient RelB knockdown in BT25 shRelB-3 cells (Figure 4G). Taken together, these data establish a critical role for RelB in driving gliomagenesis *in vivo*.

### RelB is a Prognostic Indicator in Glioma

**Figure 5:**
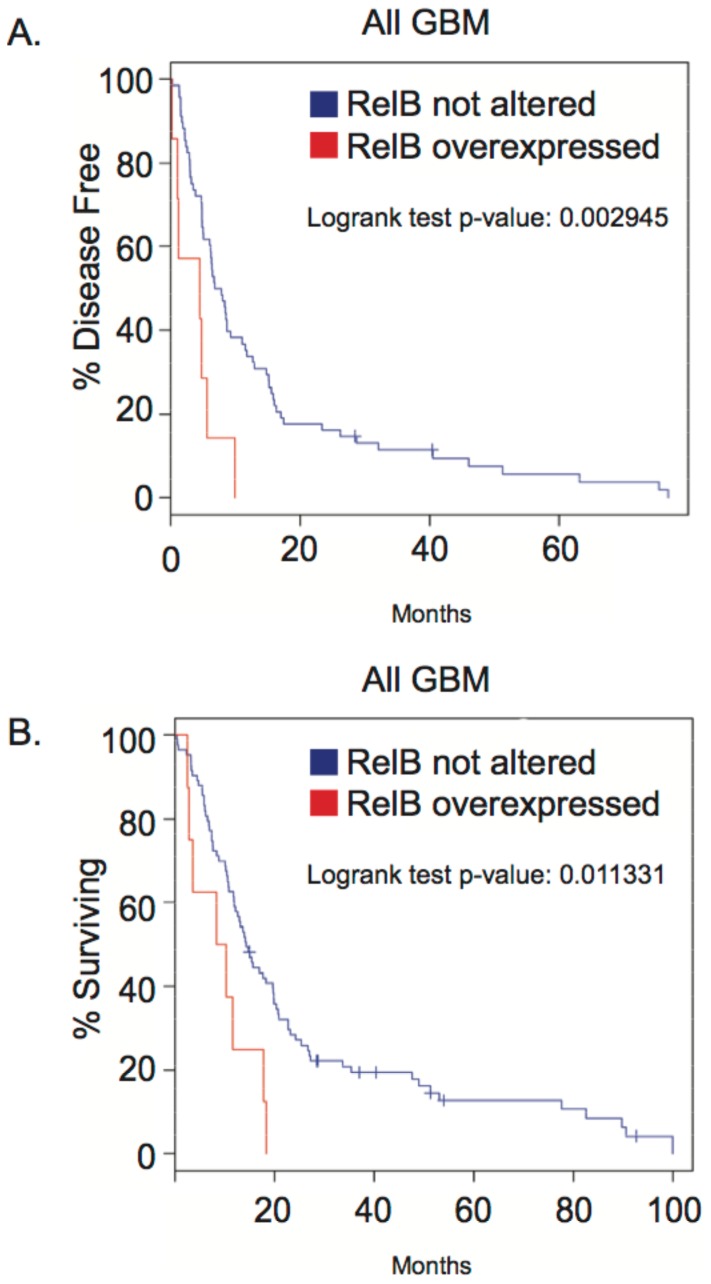
Kaplan-Meier curves from TCGA (The Cancer Genome Atlas) data analysis show the effect of RelB overexpression on time for tumor progression *(A)* and patient survival *(B)*.

Thus far, our data demonstrate that RelB is an oncogenic driver for mesenchymal glioma *in vitro* and *in vivo*. To determine whether our findings correlated with human disease, we analyzed gene expression data from The Cancer Genome Atlas (TCGA) database [26], [27]. Our analysis showed that high RelB expression in glioblastoma patients was associated with significantly shorter time to disease progression (∼10 months vs. 80 months) (Figure 5A), as well as almost 20% lower survival rates (Figure 5B). Additionally, analysis of the National Cancer Institute’s REMBRANDT (Repository of Molecular Brain Neoplasia Data) database [28] showed that increased RelB expression strongly correlates with poor survival in glioma patients, whereas low RelB expression is associated with increased survival (Supplemental Figure S1). These findings are consistent with our data demonstrating a correlation between high RelB expression and aggressive glioma pathogenesis.

## Discussion

Although glioma tumors can be broadly classified into subtypes based on gene expression profiles, the molecular mechanisms that drive tumorigenesis within each subtype are largely unknown. We have identified the NF-κB RelB protein as a strong oncogenic driver of the mesenchymal subtype of glioma and demonstrated that RelB promotes glioma cell survival, proliferation and invasiveness *in vitro* and *in vivo*. Consistent with a well-established role for RelA in positively regulating RelB expression [29], we observed a significant loss of RelB expression in RelA knockdown cells, (Supplemental Figure S2). In contrast, knockdown or overexpression of RelB did not significantly affect RelA expression or phosphorylation (Supplemental Figure S2). These results suggest that although it has previously been shown that RelA is aberrantly activated in glioma where it can promote invasion and survival [30], [31], RelB may mediate some of the tumorigenic functions attributed to RelA/p65. To-date, anti-NF-κB cancer therapy strategies have focused on targeting the canonical RelA pathway through IKKβ inhibition, with limited efficacy. However, RelA and RelB can be activated by distinct signals [8], and have distinct regulatory functions in different cell populations of a given tissue [32]. Moreover, increasing evidence suggests cross-regulation of canonical and noncanonical NF-κB signaling [33], [34]. Our results suggest that inhibition of the noncanonical RelB pathway will target cancer cell subtypes within a tumor that may be unresponsive to RelA inhibition and, consequently, may be part of a more effective treatment strategy for mesenchymal glioma.

RelB controls EMT and the expression of the mesenchymal genes in glioma, including *YKL-40/CHI3L1* (Figure 3B), a secreted glycoprotein that is a prognostic indicator for poor survival in high-grade glioma as well as other cancers and inflammatory diseases [16], [21], [35]. YKL-40 has been shown to promote glioma proliferation and invasiveness [16], and its expression is significantly increased in high-grade gliomas, as well as recurrent gliomas that exhibit a shift to a mesenchymal subtype [2]. Intriguingly, while RelB positively regulates YKL-40 (Figure 3B), our preliminary data indicate that loss of RelA does not affect basal YKL-40 expression (D.W. Lee and R. Sitcheran, unpublished data). However, RelA may be required for TNF-mediated repression of YKL-40 [36]. These findings indicate that YKL-40 can be regulated independently by distinct NF-κB signaling cascades and suggests non-redundant functions of RelA and RelB in glioma.

To our knowledge, this study is the first to report RelB-dependent regulation of YKL-40 and Olig2 in glioma. Olig2 has been shown to be expressed in neural progenitor cells and glioma CSCs [23], [24], which are proposed to be self-renewing cells that promote tumor initiation and heterogeneity [37]. In addition to the proneural glioma subtype [2], Olig2 may also play a role in the mesenchymal glioma subtype. Specifically, during vertebrate CNS development, the numbers of Olig2+ OPCs is increased by loss of the tumor suppressor *neurofibromatosis 1* (*NF1)* [38]–[40]. Since loss of NF1 is a hallmark of mesenchymal glioma [2], [12], it is possible that an increased subpopulation of Olig2^+^ cells may be a driver of oncogenesis in that tumor subtype. Indeed, Olig2-expressing (Olig2+) oligodendrycyte precursor cells (OPCs) may be candidate cells of origin in a mouse model of glioma [41], as well as human diffuse intrinsic pontine glioma (DIPG) [42], an aggressive and almost universally fatal childhood brain tumor.

In summary, our work highlights an important need to understand how specific NF-κB signaling pathways contribute to tumor initiation and progression in different tumor subtypes, as well as in heterogeneous cell populations within a given tumor. The newly identified role of RelB as an important oncogenic driver of mesenchymal glioma provides a rationale for targeting the noncanonical NF-κB signaling pathway in high RelB-expressing tumors or tumor subtypes.

## Materials and Methods

### Cells

U87-MG (U87) and 293T cells were obtained directly from ATCC and cultured in either DMEM+10%FBS and 1X Pen/Strep or Neural Stem Cell (NSC) medium (Neurobasal-A Medium, B-27 Supplement Minus Vitamin A, 1X Glutamax, 20 ng/ml EGF, 20 ng/ml bFGF and 1X Pen/Strep). All cell culture reagents are from Life Technologies, Grand Island, NY. BT cells obtained from human glioma patients (BT12, BT25, BT46, BT114) were described previously [25] and cultured in NSC medium.

### Plasmids

pLenti6-mRELB was generated by subcloning mRELB cDNA from mRelB-cFlag-pcDNA3 (Addgene) into pLenti6-V5-DEST (Life Technologies) using the GATEWAY**™** Cloning System. pLenti6-hRELB was constructed by subcloning hRELB cDNA from pCMV-Sport6-RELB (ATCC, Manassas, VA) into pLenti6-V5-DEST by GATEWAY™ Cloning System. Either pLenti6-V5-DEST without the Gateway cassette or tagRFP cloned into pLeni6-V5-DEST was used as controls for mRelB and hRelB overexpression.

### Lentivirus Production and Transduction

Mission**™**lentiviral shRNA plasmids for RelB were purchased from Sigma-Aldrich. 293T cells were transfected with 3 µg of lentiviral plasmids using polyethyleneimine (Polysciences Inc.). Lentiviruses were harvested after 3 days and used to infect 2×10^5^ glioma cells. Transduced cells were selected for 72 h in NSC medium containing 0.2 µg/ml Puromycin or 2 µg/ml Blasticidin (Invivogen, San Diego, CA).

### Real-Time RT-PCR

Total RNAs were extracted from cells using Purelink**™** RNA Mini Kit (Invitrogen) and cDNA was synthesized from 2 ug of total RNA using SuperScript® III Reverse Transcriptase (Life Technologies) following manufacturer’s instructions. Quantitative RT-PCR was performed using SYBR® Green PCR Master Mix (Applied Biosystems/Life Technologies). Expression of mRNA was normalized to either *GAPDH* or Actin expression levels. Following primers were used in amplifications; *GAPDH*, 5′- CAGGGCTGCTTTTAACTCTGG-3′, 5′- TGGGTGGAATCATATTGGAACA-3′; *RELB*, 5′- AGGCAGTCACCTCCACCTC-3′, 5′-AGCATCCTTGGGGAGAGC-3′, *TGFB3*, 5′-CACATTGAAGCGGAAAACCT-3′, 5′-AAATTCGACATGATCCAGGG-3′; *SMAD2*, 5′-TTCTTACCAAAGGCAGCA-3′, 5′-CATCGGAAGAGGAAGGAACA-3′; *OCLN*, 5′-TAGTCAGATGGGGGTGAAGG-3′, 5′-CATTTATGATGAGCAGCCCC-3′; for *BCL2*, 5′-GAGAAATCAAACAGAGGCCG-3′, 5′-CTGAGTACCTGAACCGGCA-3′, *cFLIP*, 5′-TCAGAATCCTTTCCAGTGGG-3′, 5′-CTTTGCCTCCATCTTGGGT-3′. Expression of *ACTIN, CDH1, CDH2, OLIG2, YKL-40/CHI3L1* and *RELB* expression were analyzed by using Taqman® probes (Integrated DNA Technologies (IDT), Coralville, IA). Sequence of Taqman® probes will be provided upon request. All experiments were performed at least three times with three replicates per sample.

### Quantitative Western Blots

Whole cell lysates were obtained using RIPA buffer. 25 µg of protein was separated in NuPAGE® Bis-Tris Gels (Life Technologies) and transferred onto nitrocellulose. After transfer, gels were stained with Coomassie and imaged with the IR700 channel of an Odyssey Infrared Imaging system (LI-COR Biosciences, Linclon, NE) as one check for protein loading. Two-color westerns were performed by co-incubation of one mouse and one rabbit antibody and simultaneous detection with goat anti-rabbit IRDye800CW and goat anti-mouse IRDye680 secondary antibodies (LI-COR Biosciences). Western blots and Coomassie-stained gels were scanned with the Odyssey Imager. Color images were converted to black and white and quantification of western blot data was performed using the LI-COR Image Studio software and protein levels were normalized to Tubulin, Actin and/or GAPDH. The following antibodies were used: RelB (CST-4922), RelA/p65 (SC-8008), β-Actin (SC-69879), Tubulin (SC-9104), Smad2 (CST-3103), N-Cadherin (BD-610921), YKL-40 (Bioo Scientific, 3513-04), Olig2 (SC-48817), and GAPDH (SC-32233).

### Proliferation and Cell Death Assays

For proliferation and apoptosis assays, cells were seeded at 10^4^ cells/well in 96-well plates with 200 µl NSC medium. Four replicates were prepared for each cell type and for each time point. To measure proliferation, the Cell-titer 96 Aqueous One Solution Cell Proliferation Assay MTS reagent (Promega, Madison, WI) was used according to manufacturer’s guidelines; after addition of MTS reagent and a 1 h incubation at 37C, absorbance was read at 490 nm using a VictorX3 96-well plate reader (Perkin Elmer, Waltham, MA). To measure apoptotic cell death, the Caspase-Glo 3/7 Assay system (Promega) was used according to manufacturer’s guidelines and luminescence was read using the VictorX3 96-well plate reader.

### Motility and Invasion Assays

For motility assays, 2×10^5^ cells were seeded in each well of a 24-well plate in MEM medium (10% FBS, 1X Pen/Strep, 1X Glutamax, 1X MEM-Non-essential Amino Acids and 100 mM NaPyruvate) to promote adherent growth. Cell monolayers were scratched with a pipette tip (∼1 mm wide) and photographed after 20–24 hours using bright field illumination with a 10× objective on an Olympus BH-2 microscope. Invasion assays were performed as described previously [19]. Briefly, cells were allowed to invade collagen I matrices prepared in 96-well plates. After 20–24 hours, invading cells were fixed in 3% glutaraldehyde and stained with 0.1% toluidine blue. To quantify invasion density, invading cells observed immediately below the monolayer were imaged using bright-field illumination, as described above, and numbers in equivalent fields (n = 3) were counted. Invasion distance was measured from the cell monolayer to the point of deepest penetration into the collagen matrix using cross-sectional digital images taken with an Olympus CKX41 inverted microscope and Q-Color 3 camera.

### Xenograft Mouse Models

All animal experiments were done in compliance with IACUC, AAALAC and Texas A&M Health Science Center Biosafety guidelines using an IACUC-approved Animal Use Protocol #2009-205. For subcutaneous tumor inoculations, 4–6 week old CD-1 nude mice were subcutaneously injected in the flank with 1×10^6^ cells in 100 µl 1X PBS. Animals were monitored regularly for tumor growth and sacrificed when tumor size reached ∼10 mm. For orthotopic tumor inoculations, cells were labeled using a DiD Cytoplasmic Membrane dye (Biotium, abs/em = 644/665). 5×10^5^ cells in 5 µl 1X PBS were injected into the right striatum of 4–6 week old CD-1 nude mice**.** Tumor formation was imaged using an In Vivo FX Imaging System (Carestream, Rochester, NY). Three sets of injections were done using 3 animals for each cell type (total n = 9 for each cell type). For histological analysis, tumor-bearing mice were perfused with PBS and 4% paraformaldehyde (PFA), brains were dissected and post-fixed o/n in 4% PFA, incubated in 30% sucrose o/n, and frozen in OCT for cryostat sectioning. H&E staining was performed on 10 µM sections. KI67 antibody staining (Abcam, ab16667) was visualized with an HRP conjugated ABC kit (VECTASTAIN, Vectorlabs) according to the manufacturer guidelines.

### Statistical Analyses

Student’s *t*-test was used to analyze all data except the invasion distance (Figure 3) and the real time PCR data, where a two group Kruskal Wallis signed rank test was used. Real time PCR data was evaluated by comparing delta CT values between control and experimental populations. For Figure 4B, expression levels of E-cadherin and Occludin are too low for accurate statistical analysis, but expression patterns were consistent in multiple experiments (n≥3). Equality of variances for all data was checked using O’Brien’s test, the Brown-Forsythe Test, and Bartlett’s test. The software used for these analyses was JMP 9.0.0 ©SAS 2010**.**

## Supporting Information

Figure S1 RelB is a prognostic indicator in glioma patients. Kaplan Meier curves show that increased RelB expression (A) or copy number (B) is associated with diminished survival of all glioma patients in REMBRANDT database (Repository of Brain Neoplasia Data).
(TIFF)

Figure S2 RelA/p65 knockdown results in loss of RelB, but RelB knockdown or overexpression does not affect RelA/p65 expression or phosphorylation. A) Western blot analysis was performed on U87 cells expressing control shRNA (shControl), RelB-3 or RelA/p65 shRNAs using the indicated antibodies. (B) Western blot analysis was performed on wild type U87 cells expressing pLenti6 vector, hRelB or mRelB. (C) Western blot analysis was performed on U87 cells expressing shControl or shRelB-1,-2, and -3. (D) Western blot analysis was perfomed on shRelB-1 and shRelB-3 cells that ectopically express either tagRFP vector control or mRelB.
(TIFF)

Figure S3 RelB controls cell growth, survival and invasion in BT25 cells. *(A)* MTS assays performed on BT25 shRNA control, shRelB-3 and shRelB-4 cell lines. Error bars indicate standard deviation (SD), n = 3. *(B)* A Bioluminescent assay to measure Caspase 3/7 activity was performed on BT25 cells expressing the indicated shRNA constructs. Error bars indicate SD. (C*)* Representative photographs of side views of BT25 shControl and shRelB-3 cells invading three-dimensional collagen matrices. *(D)* Average numbers of invading cells per field from 3 independent fields (+/− SD). *(E)* Quantitative real-time PCR (qRT-PCR) was performed to analyze expression of *YKL-40* and *Olig2* in BT25 shControl and shRelB-3 cells (n = 3). (*F*) Western blot analysis was performed on BT25 shControl and shRelB-3 cells with the indicated antibodies.
(TIF)
