# Supplementary figures and images for: The NF-κB RelB Protein Is an Oncogenic Driver of Mesenchymal Glioma
Source: PLoS One. 2013 Feb 25;8(2):e57489. doi: 10.1371/journal.pone.0057489 (PMC3581451; doi:10.1371/journal.pone.0057489)

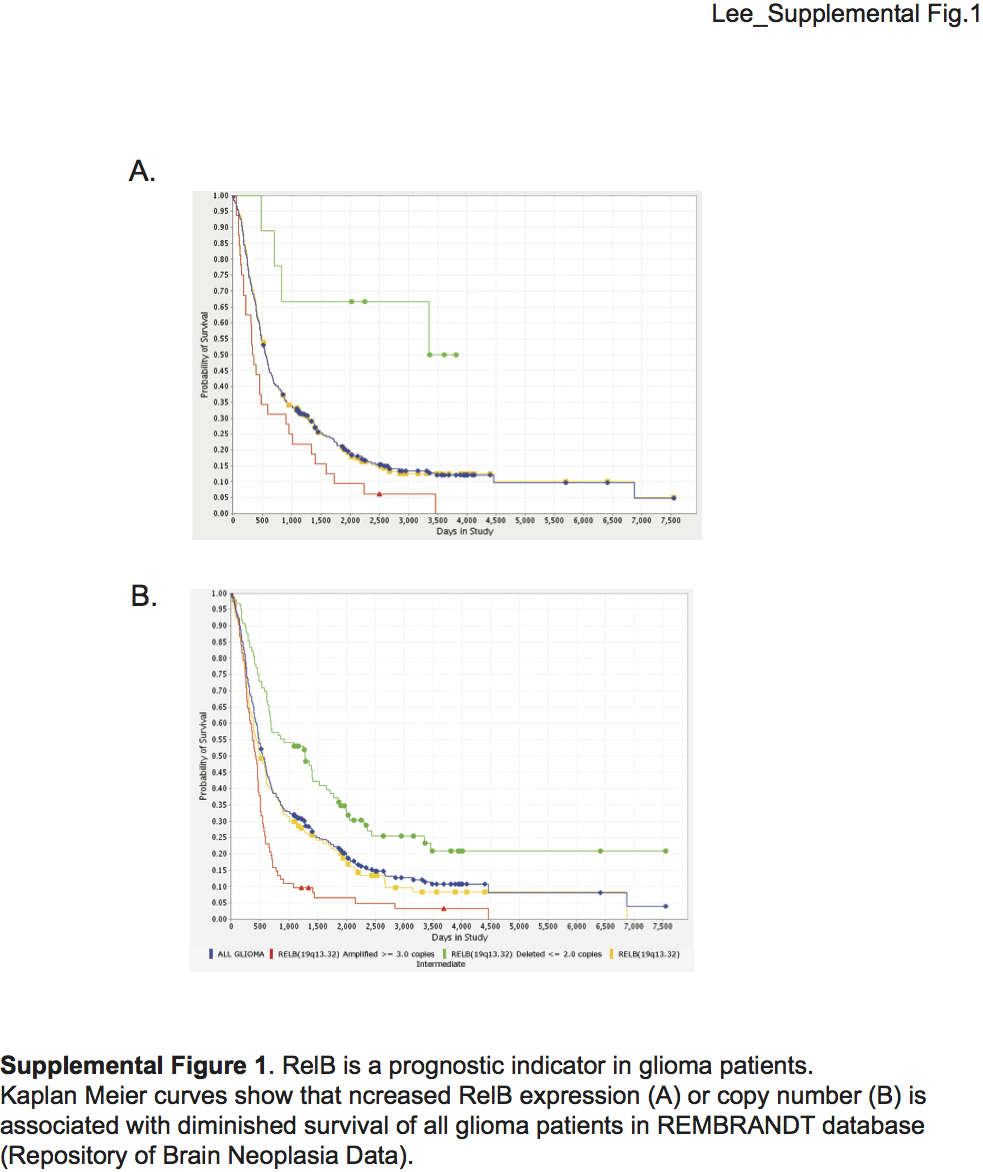

Supplement: Figure S1 — RelB is a prognostic indicator in glioma patients. Kaplan Meier curves show that increased RelB expression (A) or copy number (B) is associated with diminished survival of all glioma patients in REMBRANDT database (Repository of Brain Neoplasia Data). (TIFF) [file pone.0057489.s001.tif]

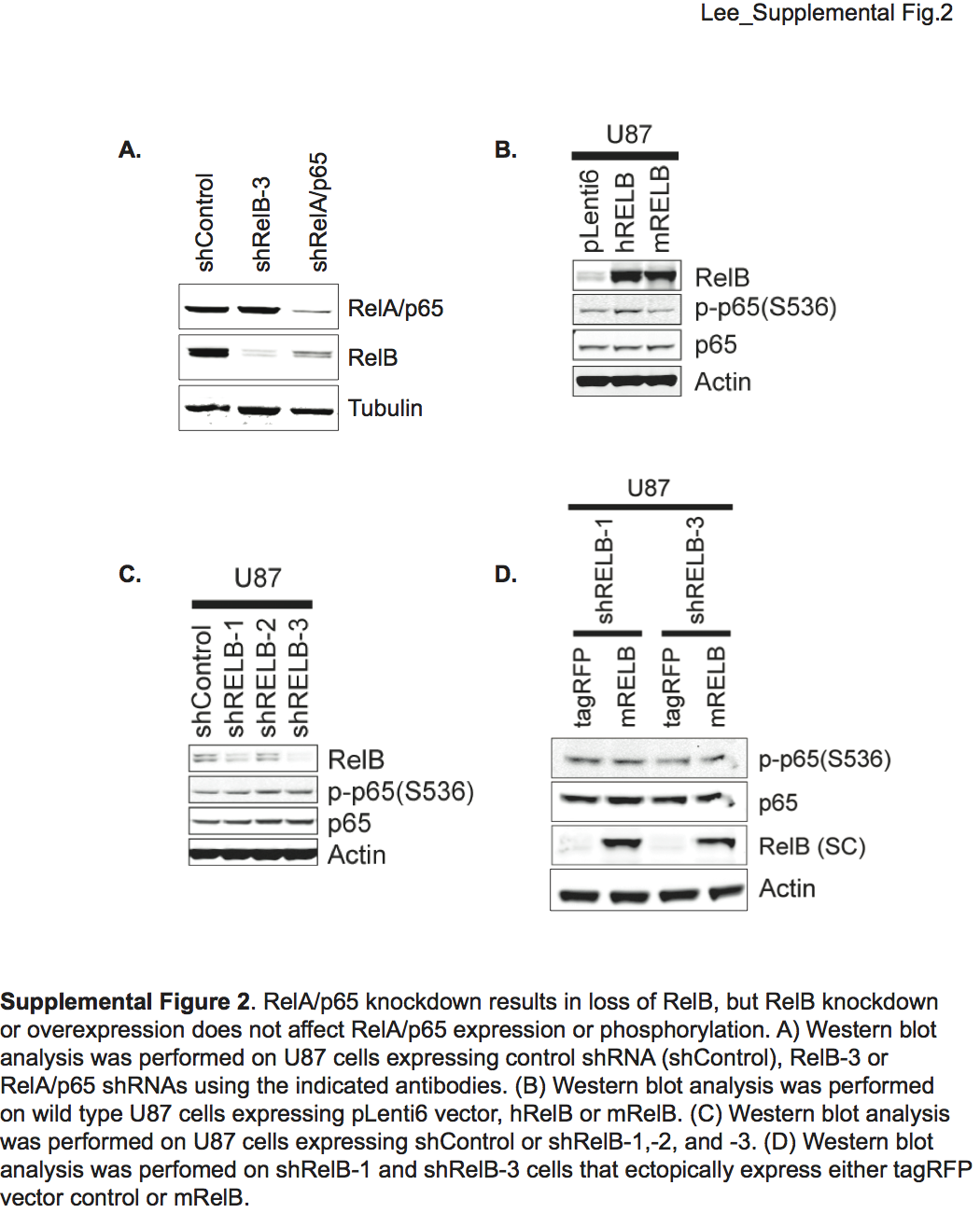

Supplement: Figure S2 — RelA/p65 knockdown results in loss of RelB, but RelB knockdown or overexpression does not affect RelA/p65 expression or phosphorylation. A) Western blot analysis was performed on U87 cells expressing control shRNA (shControl), RelB-3 or RelA/p65 shRNAs using the indicated antibodies. (B) Western blot analysis was performed on wild type U87 cells expressing pLenti6 vector, hRelB or mRelB. (C) Western blot analysis was performed on U87 cells expressing shControl or shRelB-1,-2, and -3. (D) Western blot analysis was perfomed on shRelB-1 and shRelB-3 cells that ectopically express either tagRFP vector control or mRelB. (TIFF) [file pone.0057489.s002.tif]

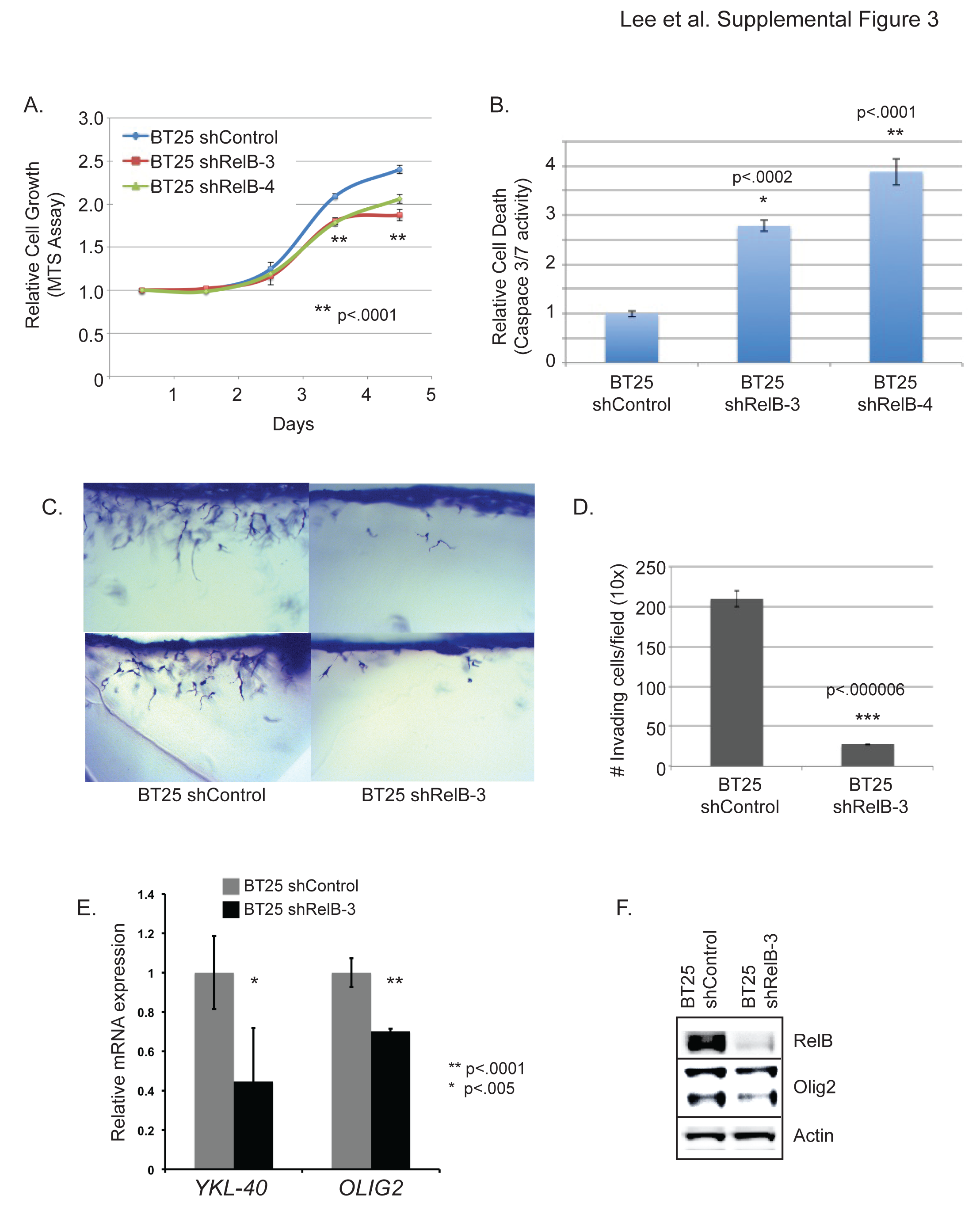

Supplement: Figure S3 — RelB controls cell growth, survival and invasion in BT25 cells. (A) MTS assays performed on BT25 shRNA control, shRelB-3 and shRelB-4 cell lines. Error bars indicate standard deviation (SD), n = 3. (B) A Bioluminescent assay to measure Caspase 3/7 activity was performed on BT25 cells expressing the indicated shRNA constructs. Error bars indicate SD. (C) Representative photographs of side views of BT25 shControl and shRelB-3 cells invading three-dimensional collagen matrices. (D) Average numbers of invading cells per field from 3 independent fields (+/− SD). (E) Quantitative real-time PCR (qRT-PCR) was performed to analyze expression of YKL-40 and Olig2 in BT25 shControl and shRelB-3 cells (n = 3). (F) Western blot analysis was performed on BT25 shControl and shRelB-3 cells with the indicated antibodies. (TIF) [file pone.0057489.s003.tif]
